# Increased Microtubule Growth Triggered by Microvesicle-mediated Paracrine Signaling is Required for Melanoma Cancer Cell Invasion

**DOI:** 10.1158/2767-9764.CRC-22-0010

**Published:** 2022-05-18

**Authors:** Karoline Pudelko, Angela Wieland, Magdalena Hennecke, Markus Räschle, Holger Bastians

**Affiliations:** 1 Institute of Molecular Oncology, Section for Cellular Oncology, Georg-August University Göttingen, University Medical Center Göttingen (UMG) and Göttingen Center for Molecular Biosciences (GZMB), Göttingen, Germany.; 2 Department of Molecular Genetics, Technical University of Kaiserslautern, Kaiserslautern, Germany.

## Abstract

The acquisition of cell invasiveness is the key transition from benign melanocyte hyperplasia to aggressive melanoma. Recent work has provided an intriguing new link between the presence of supernumerary centrosomes and increased cell invasion. Moreover, supernumerary centrosomes were shown to drive non–cell-autonomous invasion of cancer cells. Although centrosomes are the principal microtubule organizing centers, the role of dynamic microtubules for non–cell-autonomous invasion remains unexplored, in particular, in melanoma. We investigated the role of supernumerary centrosomes and dynamic microtubules in melanoma cell invasion and found that highly invasive melanoma cells are characterized by the presence of supernumerary centrosomes and by increased microtubule growth rates, both of which are functionally interlinked. We demonstrate that enhanced microtubule growth is required for increased three-dimensional melanoma cell invasion. Moreover, we show that the activity to enhance microtubule growth can be transferred onto adjacent noninvasive cells through microvesicles involving HER2. Hence, our study suggests that suppressing microtubule growth, either directly using anti-microtubule drugs or through HER2 inhibitors might be therapeutically beneficial to inhibit cell invasiveness and thus, metastasis of malignant melanoma.

**Significance:**

This study shows that increased microtubule growth is required for melanoma cell invasion and can be transferred onto adjacent cells in a non–cell-autonomous manner through microvesicles involving HER2.

## Introduction

Increased cell invasiveness is a hallmark of cancer and closely associated with metastasis (1). Cancer cell invasion involves alterations in cell shape characteristics leading to increased cell migration and the formation of actin-based invasive structures that invade into three-dimensional (3D) growth environments (2, 3). It is well known that increased cell migration and the formation of invasive structures are linked to actin reorganization that involves many regulators of actin polymerization and network organization (2, 4). In addition to the actin cytoskeleton, it is well known that also the microtubule (MT) network contributes to cell invasion (5–9). In fact, work from many laboratories has shown that inhibition of MTs using drugs that depolymerize MTs suppresses cell invasion (8, 10–12). In this context, MTs were suggested to act as cytoplasmic transport railroads and signaling platforms to modulate key actin regulators like Rho GTPases at the invasive front (7, 9, 13). More recently, it was demonstrated that not only the mere presence of MTs but rather their dynamic behavior might be important for cell invasion (14). In fact, depletion of the end-binding protein 1 (EB1), a protein that specifically associates with growing MT plus tips where it regulates MT dynamics, results in decreased cell invasion into a 3D matrix (15). In addition, inhibition of persistent MT growth either by promoting MT catastrophe events or upon depletion of the processive MT polymerase ch-TOG was shown to result in suppression of cell invasiveness (16). Thus, growing evidence suggests a pivotal role for MT dynamics in conferring invasive properties to cancer cells. This is of particular relevance also for cancer treatment because anti-MT drugs including taxanes are among the most frequently used anticancer agents and were shown to affect metastasis (10, 17, 18).

In addition to MTs, also the principal MT organizing centers, the centrosomes, have been implicated in cell invasion (19–21). Centrosomes are duplicated once during the cell cycle and this is essential to build a proper bipolar spindle apparatus with two centrosomes during mitosis, which is required for faithful chromosome segregation (22). However, centrosome alterations are frequently seen in human cancer. In particular, centrosome amplification and consequently, the presence of supernumerary centrosomes have been linked to mitotic errors through mediating abnormal spindle structures, chromosome missegregation, and aneuploidy (23, 24). Independent of that, an intriguing new link between supernumerary centrosomes and cancer cell invasion was demonstrated recently. Godinho and colleagues showed that the induction of extra centrosomes is sufficient to induce the formation of invasive structures in 3D model systems derived from breast epithelial cells (19). Moreover, it was shown that the presence of extra centrosomes as well as centrosome aberrations contribute to cell invasion in a non–cell-autonomous manner (20, 25). Here, a centrosome-induced paracrine signaling, which might involve increased secretion of soluble signaling proteins including cytokines, was suggested to contribute to the formation of invasive actin structures (20). In addition, the activation of oncogenic HER2 in the recipient cells was shown to be involved in triggering invasive characteristics upon centrosome amplification (20). Most recently, the importance of supernumerary centrosomes in cell invasion was further strengthened by the finding that pancreatic cancer cells with supernumerary centrosomes exhibit increased secretion of extracellular vesicles (EV), which trigger the activation of pancreatic stellate cells in the tumor microenvironment through a yet unknown mechanism. Importantly, these activated cells, in turn, promote cell invasion of pancreatic cancer cells in 3D models (21). These findings suggest that supernumerary centrosomes contribute to cancer cell invasion by promoting intercellular communication through secreted soluble proteins and by EVs. In particular, the latter is highly cancer relevant because EVs, which include larger microvesicles (MV) and small exosomes, have been recognized as important cell-to-cell communication routes that contribute to progression and aggressive phenotypes of cancer. EVs can horizontally transfer molecules such as proteins, DNAs, RNAs, miRNAs, lipids, and oncogenic signaling molecules to initiate profound phenotypic changes in the recipient cells including increased tumor growth and invasion capabilities (26, 27). The important role of EVs to increase cancer aggressiveness has also been described in malignant melanoma where the acquisition of invasive behavior is the key transition from benign melanocyte hyperplasia to aggressive and life-threatening melanoma (28). However, little is known about the role of centrosomes, dynamic MTs, or EVs in melanoma cell invasion.

We set out to investigate the role of supernumerary centrosomes and dynamic MTs in melanoma cell invasion. We show that highly invasive melanoma cells are characterized by supernumerary centrosomes associated with increased MT growth rates and demonstrate that both phenotypes are causally interlinked. We show that increased MT growth rates are required for invasiveness of melanoma cells in 3D models. Moreover, we revealed that supernumerary centrosomes and thus, increased MT growth, promote MV-mediated HER2 signaling that triggers increased MT growth in recipient cells to support cell invasion.

## Materials and Methods

### Cell Culture

Melanoma cell lines SK-Mel-19 (RRID:CVCL_6025), SK-Mel-173 (RRID:CVCL_6090), SK-Mel-103 RRID:CVCL_6069), and SK-Mel-147 (RRID:CVCL_3876) were kindly provided by Maria S. Soengas (Spanish National Cancer Research Center, Madrid, Spain). All cell lines were short tandem repeat profiled, regularly tested for *Mycoplasma* using PCR, and maintained in RPMI1640 (PAN-Biotech GmbH) supplemented with 10% FCS (Corning), 100 μg/mL streptomycin, and 100 units/mL penicillin (Anprotec) at 37°C and 5% CO_2_ for up to 1 month.

### Conditioned Medium and MVs

To obtain conditioned medium (CM), cells were counted and 14 × 10^6^ cells were cultured for 24 hours in a 10 cm dish with 5 mL RPMI1640 without phenol red (PAN-Biotech) supplemented with 1% penicillin-streptomycin. Medium supernatant was collected and centrifuged at 2,000 × *g* for 10 minutes to remove cells and cellular debris. The supernatant was diluted (60:40) with fresh medium and used to treat recipient cells. To isolate larger MVs, the supernatant was centrifuged for 35 minutes at 14,000 × *g*. The pellet was resuspended in growth medium and used for treatment of recipient cells.

### Coculture

A total of 0.2 × 10^6^ SK-Mel-173 cells were transfected pEGFP-EB3 plasmid (kindly provided by L. Wordeman) using electroporation and cocultured with an equal number of untransfected SK-Mel-173 or SK-Mel-103 cells in a 6-well plate. On the basis of an equal splitting ratio during cell maintenance, the different cell lines exhibit similar growth kinetics. After 24 hours, the growth rates of MTs were determined by live-cell microscopy detecting EB3-EGFP comets.

### 3D Invasion

A total of 500 cells were seeded into Nunclon U-bottom ultra-low attachment 96-well plates (Thermo Fisher Scientific) and centrifuged at 874 × *g* for 20 minutes at room temperature. After 48 hours, spheroids were transferred to Matrigel (10 mg/mL, Corning) diluted with growth medium in a ratio of 70:30. After solidification for 30 minutes at 37°C, the gel matrix containing the spheroids was overlayed with growth medium and incubated at 37°C. Images of spheroids were taken after 48 or 72 hours. The area of spheroid outgrowth was measured using ImageJ software (NIH Image, RRID:SCR_003070) and normalized to the area of control spheroids.

### Inhibitor Treatments

Cells were treated either with 0.5 nmol/L Taxol (Sigma-Aldrich), 40 μmol/L trastuzumab (reversible HER2 inhibitor, kindly provided by Gerald Wulf) or 200 nmol/L canertinib (irreversible HER2 kinase inhibitor, Selleck Chemicals). For live-cell microscopy experiments, cells were treated with inhibitors for 16 hours. Treatment of cell spheroids started 24 hours before transferring spheroids to Matrigel.

### Transfection

Cells were transfected with siRNAs using ScreenFect according to the manufacturer's protocol. The following unmodified siRNAs were custom made and purchased from Sigma:

*LUCIFERASE (LUC)*: 5′ CUU ACG CUG AGU ACU UCG AUU- 3′
*CKAP5*: 5′ -GAG CCC AGA GTG GTC CAA A- 3

Cells were transfected with plasmids by electroporation using a Bio-Rad Genepulser Xcell device (950 μF, 220 V, ∞ Ω‘, 4 mm cuvette) or by using ScreenFect A or Lipofectamine 3000 (Invitrogen) according to the company’s instructions. To detect growing MT plus ends, cells were transfected with pEGFP-*EB3* or pmCherry-*EB3* (kindly provided by Linda Wordeman). To overexpress *PLK4*, pCMV-Flag-*PLK4* (kindly provided by Ingrid Hofmann) was used. To generate SK-Mel-103 cells with stable knockdown of ch-TOG, cells were stably transfected with pLKO1-*CKAP5* short hairpin RNA vector (29) or control plasmid and single-cell clones were selected in medium containing 10 μg/mL blasticidin.

### Immunofluorescence Microscopy

For staining of centrosomes, cells were fixed with 2% paraformaldehyde for 5 minutes at room temperature followed by permeabilization with methanol for 5 minutes at −20°C. After blocking with PBS supplemented with 5% FCS for 30 minutes at room temperature, cells were incubated overnight at 4°C with the staining solution containing the primary antibody (γ-tubulin, clone GTU88, #T6557, Sigma-Aldrich, RRID:AB_477584) diluted in PBS supplemented with 2% FCS. Incubation with the secondary antibody (Alexa-Fluor 594, anti-mouse, Invitrogen) in PBS supplemented with 2% FCS was done for 1.5 hours and was followed by DNA staining using Hoechst33342.

### Determination of MT Plus End Growth Rates

To determine MT plus end growth rates, cells were transfected by electroporation with 10 μg pEGFP-*EB3* or pmCherry-*EB3* as described previously (29). A total of 48 hours after transfection, live-cell microscopy of interphase cells was performed at 37°C and 5% CO_2_ using a Delta Vision Elite live-cell microscope (Applied Precision) as described previously (29). Images were acquired every 2 seconds for 30 seconds in total with an optical z-stack distance of 0.4 μm.

### FACS Analysis

To determine cell-cycle progression and cell death, FACS analysis was performed using a BD FACS Canto II flow cytometer (Becton Dickinson) as described previously (29).

### Western Blotting

Cells were lysed in lysis buffer [50 mmol/L Tris-HCl pH 7.4, 150 mmol/L NaCl, 5 mmol/L EGTA pH 8.0, 5 mmol/L EDTA pH 8.0, 1% (v/v) NP40, 0.1% (w/v) SDS, 0.1% (w/v) sodium deoxycholate, phosphatase inhibitor cocktail (25 mmol/L β-glycerophosphate, 50 mmol/L NaF, 5 mmol/L Na_2_MoO_4_, 0.2 mmol/L Na_3_VO_4_, 5 mmol/L EDTA, 0.5 μm microcystin], complete EDTA-free protease inhibitor cocktail (Roche). A total of 8% or 10% SDS polyacrylamide gels were prepared and proteins were separated followed by the transfer to nitrocellulose membranes. The following antibodies were used: anti-α-tubulin (B-5-1-2, 1:1,000, Santa Cruz Biotechnology, #sc-23948, RRID:AB_628410), anti-β-actin (AC-15, 1:10,000, Sigma-Aldrich, #A5441, RRID:AB_476744), anti-chTOG (H-4, 1:400, Santa Cruz Biotechnology, #sc-374394, RRID:AB_10987687), anti-Plk4 (6H5, 1:400, Merck Millipore, #MABC544, RRID:AB_2893410), anti-STIL (1:5,000, Bethyl Laboratories, #A302442A, RRID:AB_1944269), anti-HER2 (1:1,000, Cell Signaling Technology, #2242, RRID:AB_331015), anti-mouse/rabbit-HRP (1:10,000, Jackson ImmunoResearch, #115-035-146, #111-035-144, RID:AB_2307392). All Western blots were performed from at least three independent experiments.

### Mass Spectrometry

For mass spectrometry (MS) analysis, MVs were isolated from medium supernatant from noninvasive SK-Mel-173 cells with or without *PLK4* overexpression as described above. MVs were resuspended in lysis buffer [6 mol/L guanidinium chloride, 10 mmol/L tris (2-carboxyethyl) phosphine, 40 mmol/L CAA chloroacetamide, 100 mmol/L Tris pH 8.5] and incubated for 10 minutes at 96°C. Samples were sonicated 5 minutes in a sonication water bath (Bandelin Sonorex) and protein concentration was determined using a BCA assay (Thermo Fisher Scientific). A total of 25 μg of protein was diluted 10-fold with digestion buffer (10% acetonitrile, 25 mmol/L Tris pH 8.5) was digested with LysC (1:50 w/w, Wako) and trypsin (1:50 w/w, Sigma) for 16 hours at 37°C. Soluble peptides were desalted, samples were vacuum dried, and resolubilized in buffer A (0.1% formic acid in MS grade water). Peptides were separated by nano-high-pressure liquid chromatography on an Easy nLC 1200 chromatography system (Thermo Fisher Scientific) and sprayed directly into a Q Exactive HF mass spectrometer (Thermo Fisher Scientific). For this, peptides were injected onto a 50 cm column with an inner diameter of 75 μm packed in-house with C18 beads (ReproSil-Pur 120 C18 AQ 1.9 μm, Dr. Maisch). Peptides were eluted with a 3-hour gradient using a mixture of buffer A (0.1% formic acid) and buffer B (80 acetonitrile, 0.1% formic acid) ranging from 2% to 95% buffer B. MS data were acquired using a top 15 method. MS data were processed using MaxQuant Version 1.6.17.0 (RRID:SCR_014485) and the Perseus software Version 1.6.14.0 (ref. 30; RRID:SCR_015753). To identify proteins enriched in MVs from SK-MEL-173 cells overexpressing *PLK4* compared with cells transfected with the control vector, label-free quantified intensities were filtered to contain at least five valid values in total. Missing values were imputed and a modified *t* test implemented in Perseus with a permutation-based FDR control was used to call significantly upregulated or downregulated proteins (FDR = 0.05, S0 = 1) as described previously (30).

### Statistical Analysis

For robust calling of significantly deregulated proteins in our MS analysis, we used permutation-based estimation of the FDR according to Tusher and colleagues (31), which has been implemented into the Perseus software (30). In addition, we have also calculated the adjusted *P* values according to the procedure proposed by Benjamini and Hochberg and added the adjusted *P* value to the Supplementary Table S1 (column D).

For all other data types, GraphPad Prism 5.0 software (GraphPad Software; RRID:SCR_002798) was used for statistical analysis. Mean values and SD were calculated. Unpaired two-tailed *t* tests (SD ≠ 0) or one-sample *t* tests (SD = 0) without multiple testing were applied to analyze statistical significance. *P* values were indicated as: ns (not significant): *P* ≥ 0.05; *, *P* < 0.05; **, *P* < 0.01; ***, *P* < 0.001; ****, *P* < 0.0001.

### Data Availability

The MS proteomics data have been deposited to the ProteomeXchange Consortium via the PRIDE (32; RRID:SCR_012052) partner repository with the dataset accession number PXD032224.

## Results

### Supernumerary Centrosomes and Increased MT Growth Rates are Associated with Invasiveness of Melanoma Cells

**FIGURE 1.**
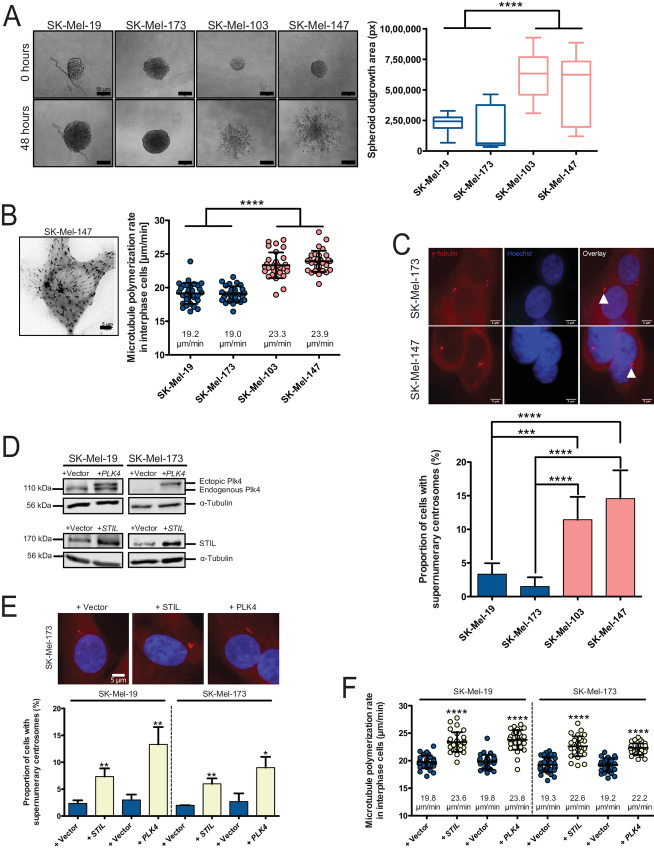
Supernumerary centrosomes and increased MT growth rates are associated with invasiveness of melanoma cells. **A,** 3D invasion of melanoma cell lines. Spheroids of the indicated melanoma cell lines were embedded into Matrigel and 3D invasion was followed for 48 hours. Left: Representative images of spheroids and invaded cells. Scale bar, 50 μm. Right: Measurements of the spheroid outgrowth area after 48 hours in Matrigel (mean ± SD, *n* = 19–23 spheroids, *t* test). **B,** Live-cell measurements of MT plus end growth rates in melanoma cells during interphase. Left: Example image of a melanoma cell expressing EB3-GFP and used for measurements of MT growth rates by live-cell microscopy. Right: Determination of MT growth rates in the indicated melanoma cell lines. Scatter dot plots showing average MT growth rates (20 MTs/cell, *n* = 30 cells, mean ± SD, *t* test). **C,** Detection of supernumerary centrosomes in invasive and noninvasive melanoma cells. Top: Representative immunofluorescence microscopy images of melanoma cells with or without supernumerary (>2) centrosomes and analyzed by detecting γ-tubulin as a marker for centrosomes. White triangles mark centrosomes, nuclei were stained by Hoechst. Scale bar, 5 μm. Bottom: Quantification of the proportion of cells with supernumerary centrosomes (mean ± SD, *n* = 600 cells, *t* test). **D,** Representative Western blots detecting *PLK4* or *STIL* overexpression in noninvasive melanoma cells. α-tubulin was detected as a loading control. **E,** Quantification of the proportion of noninvasive melanoma cells with supernumerary centrosomes upon *STIL* or *PLK4* overexpression. Top: example images of SK-Mel-173 cells with or without *STIL* or *PLK4* overexpression. Centrosomes were stained by anti-γ-tubulin antibodies and nuclei were stained by Hoechst. Scale bar, 5 μm. Bottom: The graph shows the proportion of cells with supernumerary centrosomes (mean ± SD, *n* = 300 cells, *t* test). **F,** Determination of MT plus end growth rates in the indicated noninvasive melanoma cells after *PLK4* or *STIL* overexpression. Scatter dot plots showing average MT growth rates (20 MTs/cell, *n* = 30 cells, mean ± SD, *t* test).

We aimed to investigate the role of centrosomes and dynamic MTs in melanoma cell invasion. To this end, we used melanoma cell lines previously characterized as being invasive or noninvasive (33–35). First, we verified the invasive status of the cells by following the formation of invasive protrusions of cell spheroids into a 3D Matrigel matrix. As anticipated, SK-Mel-103 and SK-Mel-147 cells exhibited strong invasive activity while SK-Mel-19 and SK-Mel-173 cells showed only minor 3D invasion (Fig. 1A). Because growing MTs were previously shown to play a role in cancer cell invasion (9), we asked whether differential MT growth behavior might be associated with the invasiveness of melanoma cells. For this, we measured MT growth rates in invasive and noninvasive melanoma cells by live-cell microscopy detecting end-binding protein 3 (EB3)-GFP comets at MT plus tips in the cytoplasm of interphase cells (ref. 36; Fig. 1B; Supplementary Video S1). Indeed, we found a correlation between increased MT plus end growth rates and high invasiveness of melanoma cells (Fig. 1B). Given the previously reported link between increased centrosome numbers and cell invasion in breast carcinoma cells (19, 20), we also determined cells exhibiting supernumerary centrosomes (cells with > 2 centrosomes) in the melanoma cells by immunofluorescence microscopy detecting γ-tubulin as a standard marker for centrosomes (Fig. 1C, top). Significantly, increased invasion capabilities were not only associated with enhanced MT growth, but also with the presence of supernumerary centrosomes (Fig. 1C) demonstrating a correlation between increased MT growth rates, centrosome numbers, and cell invasion. It is of note that only 10%–15% of the invasive melanoma cells harbor supernumerary centrosomes (Fig. 1C) while the increase in MT growth is observed in the majority of the cells analyzed (Fig. 1B). As we show later in this work, this is likely due to a paracrine induction of increased MT growth rates.

The correlation between supernumerary centrosomes and increased MT growth rates in invasive melanoma cells prompted us to test whether supernumerary centrosomes can trigger increased MT growth rates. To this end, we induced centrosome amplification by overexpression of key regulators involved in the centrosome duplication cycle, namely, *PLK4* and *STIL* (37, 38), in noninvasive melanoma cells that show otherwise no aberrant centrosome numbers (Fig. 1D). As expected, both means significantly increased the number of centrosomes in these cell lines (Fig. 1E). Importantly, subsequent measurements of MT plus end growth rates in living interphase cells revealed that increasing centrosome numbers causes increased MT plus end growth rates (Fig. 1F) indicating a causal relationship between supernumerary centrosomes and increased MT growth rates in invasive melanoma cells. However, the molecular mechanism explaining why supernumerary centrosomes cause increased MT plus end growth remains unknown.

### Increased MT Plus End Growth Rates Rather Than Supernumerary Centrosomes Contribute to Cell Invasion in Melanoma

**FIGURE 2.**
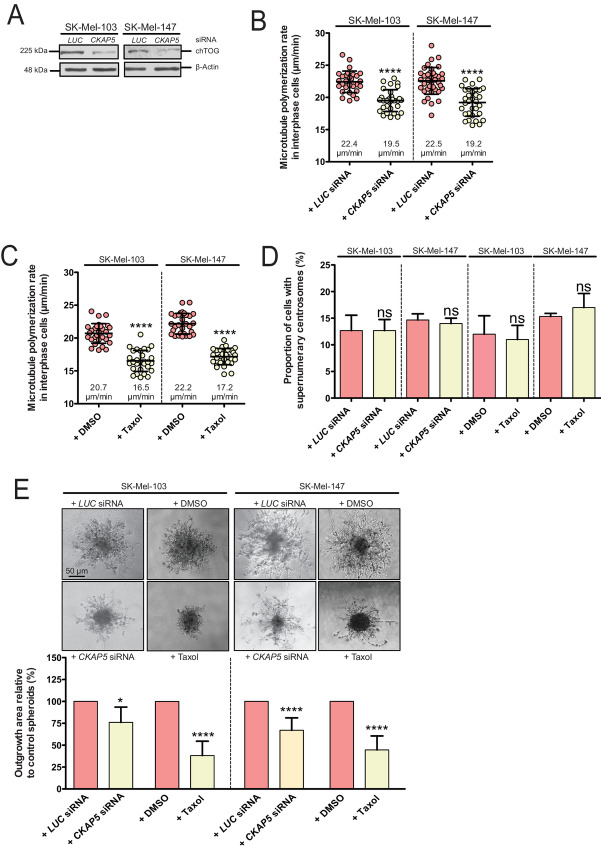
Supernumerary centrosomes cause increased MT growth rates to promote melanoma cell invasion. **A,** Representative Western blots showing siRNA mediated downregulation of ch-TOG in the indicated invasive melanoma cells. α-tubulin was detected as a protein loading control. **B,** Determination of MT plus end growth rates in invasive melanoma cell lines after partial siRNA-mediated depletion of ch-TOG. Scatter dot plots show average MT growth rates (20 MTs/cell, mean ± SD, *n* = 30, *t* test). **C,** Measurements of MT growth rates in invasive melanoma cell lines after treatment with DMSO (control) or 0.5 nmol/L Taxol for 16 hours. Noninvasive SK-Mel-173 cells were used as control. Scatter dot plots show average MT growth rates (20 MTs/cell, mean ± SD, *n* = 30, *t* test). **D,** Quantification of the proportion of invasive melanoma cells with supernumerary centrosomes upon partial depletion of ch-TOG or after treatment with Taxol (mean ± SD, *n* = 300 cells, *t*-test). **E,** Invasive 3D outgrowth of spheroids derived from invasive SK-Mel-103 melanoma cells and treated with 0.5 nmol/L Taxol or after siRNA-mediated depletion of ch-TOG. Spheroids were embedded into Matrigel and 3D outgrowth was followed for 48 hours in the presence or absence of treatments. Top: representative microscopy images showing outgrowth of 3D spheroids derived from SK-Mel-103 and SK-Mel-147 cells. Scale bar, 50 μm. Bottom: Quantification of the 3D outgrowth area of spheroids derived from the indicated invasive melanoma cells after treatment with DMSO (control), 0.5 nmol/L Taxol or upon siRNA-mediated knockdown of *CKAP5* after 48 hours (mean ± SD, *n* = 18–26 spheroids, *t* test).

Because we found that supernumerary centrosomes and increased MT growth rates are associated with increased melanoma cell invasion, we asked whether one or the other is required for cell invasion. To address this, we specifically rescued the increased MT growth rates in invasive melanoma cells without affecting centrosome numbers. This was achieved by partially depleting the MT plus end polymerase ch-TOG (39) or by treatment with subnanomolar doses of the MT dynamics inhibiting drug Taxol. Both means were previously shown to decrease MT plus end growth rates in mitosis without affecting cell viability *per se* (29, 40) and we verified that these treatments do not grossly affect cell-cycle progression or cell viability (Supplementary Fig. S1A). As expected, partial siRNA-mediated depletion of ch-TOG (encoded by *CKAP5;*Fig. 2A) rescued increased MT plus end growth rates in interphase cells (Fig. 2B) to similar levels observed in noninvasive melanoma cells (compare with Fig. 1B). Treatment with subnanomolar concentrations of Taxol lowered MT growth rates slightly more (Fig. 2C). Neither ch-TOG depletion nor Taxol treatment affected the increased centrosome numbers present in these cells (Fig. 2D) indicating that these treatments are suitable means to selectively rescue the MT growth rate abnormality in the invasive melanoma cells. Using these conditions, we determined 3D invasion of the melanoma cells and found a suppression of invasive protrusions upon lowering MT growth rates (Fig. 2E). Thus, increased MT plus end growth rates, but not supernumerary centrosomes *per se* represent a requirement for melanoma cell invasion.

Next, we asked whether increased MT plus end growth rates are not only required but also sufficient to trigger cell invasion. To this end, we overexpressed *CKAP5* to induce increased MT growth rates in noninvasive SK-Mel-19 and SK-Mel-173 melanoma cells to levels typically observed in the invasive melanoma cells (Supplementary Fig. S1B and S1C). However, subsequent 3D invasion assays showed no significant induction of 3D cell invasion and cell spreading into the Matrigel upon induction of increased MT dynamics, although a slight loosening of the cellular border was seen upon *CKAP5* overexpression (Supplementary Fig. S2A). We also investigated whether *PLK4* or *STIL* overexpression, which causes the induction of centrosome amplification and leading to increased MT growth rates (Fig. 1E and F), is inducing 3D cell invasion, but we could not observe an increase in invasion activity (Supplementary Fig. S2B). Together, these results suggest that increased MT growth rates contribute to, but are not sufficient for significant 3D melanoma cell invasion.

### Induction of MT Plus End Growth Activity by Paracrine Signaling

**FIGURE 3.**
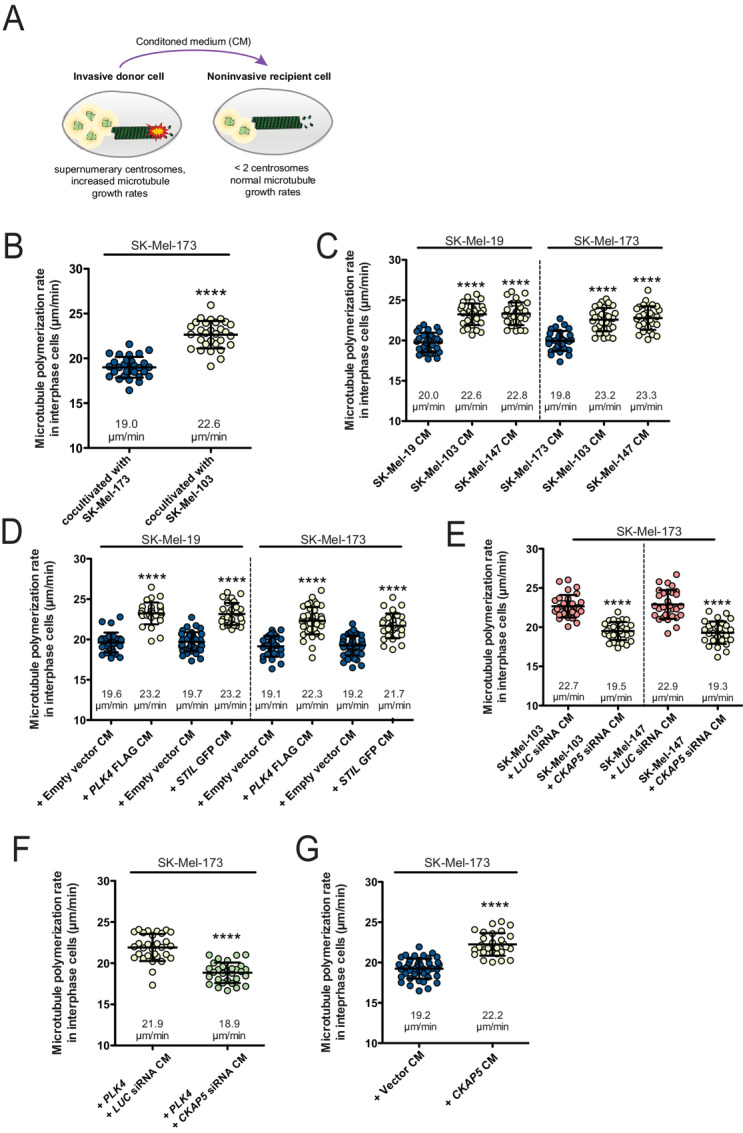
Induction of increased MT growth activity by paracrine signaling. **A,** Model depicting the paracrine induction of increased MT growth rates. **B,** Measurements of MT growth rates in noninvasive SK-Mel-173 cells after cocultivation of noninvasive or invasive melanoma cells for 24 hours. **C,** Measurements of MT growth rates in the indicated noninvasive melanoma cells after treatment with CM derived from noninvasive or invasive cells for 16 hours. **D,** Measurements of MT growth rates in the indicated noninvasive melanoma cells after treatment with conditioned media derived from the same cells with or without *PLK4* or *STIL* overexpression. **E,** Measurements of MT growth rates in noninvasive melanoma cells after treatment with conditioned media derived from the indicated invasive melanoma cells with or without partial depletion of ch-TOG (*CKAP5* repression). **F,** Measurements of MT growth rates in noninvasive SK-Mel-173 cells after treatment with conditioned media derived from the same cells with *PLK4* overexpression and concomitant *CKAP5* repression. **G,** Measurements of MT growth rates in noninvasive SK-Mel-173 cells after treatment with conditioned media derived from the same cells with or without *CKAP5* overexpression. All scatter dot plots show average MT growth rates (20 MTs/cell, mean ± SD, *n* = 30, *t* test).

Recent studies have revealed that supernumerary centrosomes can contribute to cancer cell invasion in a non–cell-autonomous manner involving paracrine signaling (20). This prompted us to investigate whether the phenotype of increased MT growth rates as an invasion contributing factor might be horizontally transferred from invasive to noninvasive cells (Fig. 3A). First evidence for such a regulation came from coculture experiments in which we cocultivated invasive with noninvasive melanoma cells and determined MT growth rates specifically in (EB3-GFP expressing) noninvasive cells. In fact, the presence of invasive SK-Mel-103 cells was sufficient to increase MT growth rates in noninvasive cells (Fig. 3B). Next, we tested whether conditioned growth medium (CM) derived from invasive cells contains an activity to increase MT growth rates in noninvasive melanoma cells. We treated noninvasive cells with CM derived from invasive cells (SK-Mel-103 or SK-Mel-147) and found an induction of increased MT dynamics in noninvasive cells within 2 hours (Fig. 3C). Similarly, when using CM derived from noninvasive cells expressing *PLK4* or *STIL* to induce supernumerary centrosomes and increased MT dynamics (Fig. 2) we found the same induction of increased MT growth rates in the recipient cells (Fig. 3D). At the same time, treatments with CM from invasive cells or from cells with *PLK4* or *STIL* overexpression did not affect centrosome numbers in the noninvasive cells (Supplementary Fig. S3A and S3B). We conclude that CM harvested from invasive melanoma cells contains an activity, which specifically induces increased MT growth rates in the recipient cells without affecting centrosome numbers.

We then investigated whether supernumerary centrosomes present in invasive cells or in cells with *PLK4* overexpression or rather increased MT growth represents the relevant trigger for the paracrine signaling responsible to transfer abnormal MT dynamics from invasive onto noninvasive cells. To this end, we partially depleted ch-TOG from invasive cells to restore low MT growth rates in the presence of supernumerary centrosomes (Fig. 2B–D), collected CM and treated noninvasive cells with the CM. In fact, lowering MT growth rates in the donor cells completely abrogated the activity to enhance MT growth rates in the recipient cells (Fig. 3E). The same result was obtained when we used CM from cells with *PLK4* overexpression and concomitant *CKAP5* depletion (Fig. 3F). In addition, we also induced MT growth rates in noninvasive melanoma cells by overexpression of *CKAP5* and used CM from these cells. In line with the *CKAP5* depletion experiments, we found that increasing MT growth was sufficient to trigger paracrine signaling to induce increased MT growth rates in the recipient cells (Fig. 3G). Together, these results indicate that increased MT growth rates act as a trigger for paracrine signaling to transfer the phenotype of abnormal MT dynamics to surrounding cells where it supports cell invasion.

### MVs Mediate Increased MT Growth Rates

**FIGURE 4.**
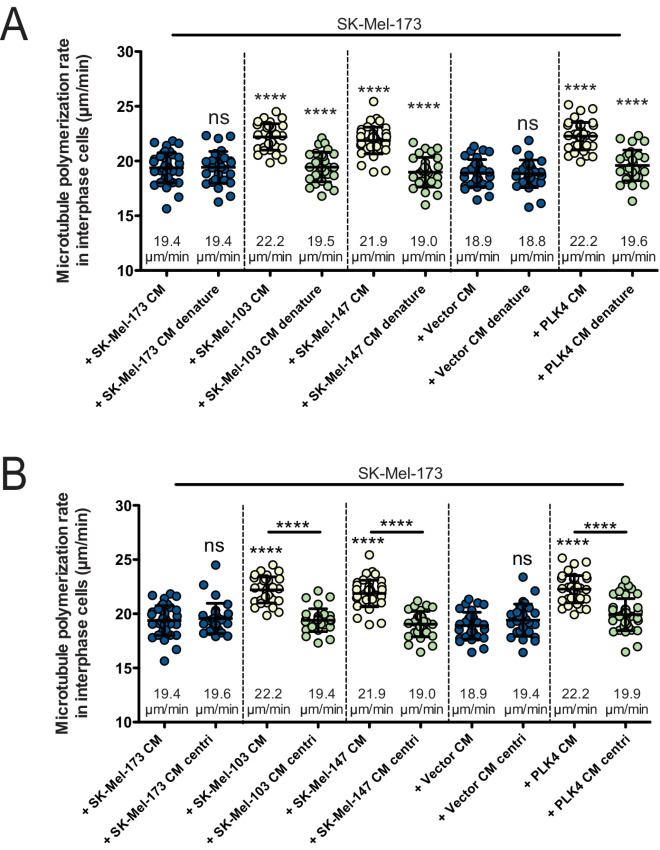
MVs mediate increased MT growth rates. **A,** Measurements of MT growth rates in noninvasive melanoma cells after treatment with CM derived from noninvasive or invasive cells before and after centrifugation to deplete MVs from the media. **B,** Measurements of MT growth rates in noninvasive melanoma cells after treatment with conditioned media derived from noninvasive or invasive cells before and after heating the media for 30 minutes at 56°C to denature proteins. All scatter dot plots show average MT growth rates (20 MTs/cell, mean ± SD, *n* = 30, *t* test).

Paracrine signaling for cell-to-cell communication can involve the secretion of soluble proteins or other molecules into the extracellular space. On the other hand, also EVs of different sizes including larger MVs and small exosomes are well known to contribute to paracrine intercellular communication, also in melanoma where they can transfer DNA, RNA, miRNA, and proteins including oncogenic factors from one cell to another (26, 28). In addition, recent studies indicated that supernumerary centrosomes can support shedding of EVs to promote cell invasion in pancreatic cancer by activating cancer-associated fibroblast-like cells (21). First, we tested whether protein denaturation would inactivate the activity that triggers increased MT growth rates in recipient cells. Indeed, heating the CM to 56°C completely abolished the activity indicating that proteins rather than other molecules might be involved in triggering MT growth rates (Fig. 4A). To test whether the phenotype of increased MT growth is transferred through soluble secreted proteins, small exosomes or larger MVs, CM from invasive melanoma cells was subjected to differential centrifugation (Supplementary Fig. S4A). As shown in Fig. 4B, centrifugation at 14,000 × *g*, which removes only MVs from CM, abrogated the induction of increased MT growth in noninvasive cells indicating that MVs rather than exosomes or soluble proteins contain the activity to increase MT growth. Thus, we suggest that protein-containing MVs rather than small exosomes or soluble secreted proteins are responsible for the paracrine signaling leading to increased MT growth in recipient melanoma cells.

### MVs Involving HER2 Mediate Increased MT Growth Rates and Cell Invasion

**FIGURE 5.**
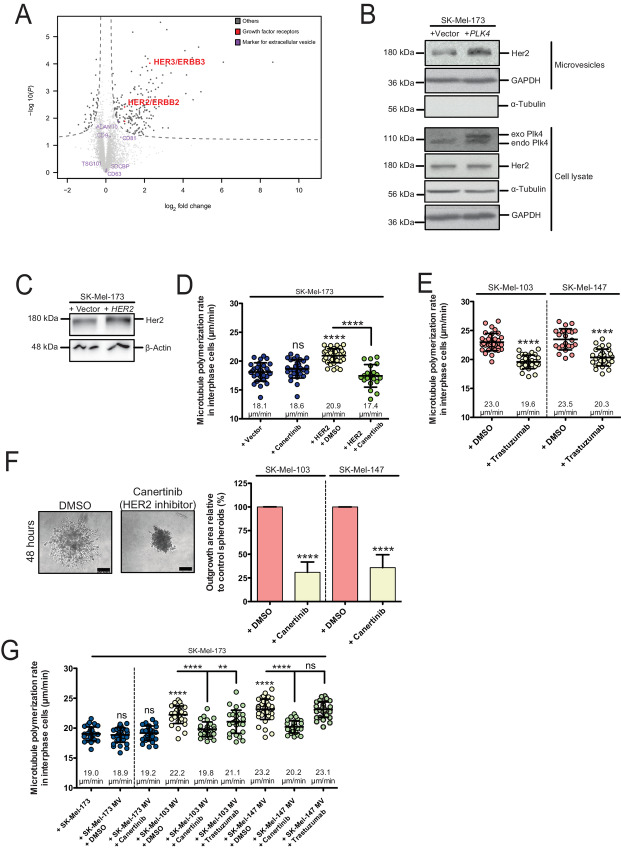
MVs comprising HER2 mediate enhanced MT growth. **A,** Identification of HER2/ERBB2 as a constituent of MVs upon induction of supernumerary centrosomes. Isolated MVs derived from noninvasive SK-Mel-173 cells with or without overexpression of *PLK4* were subjected to MS analysis. The volcano plot depicts HER2/ERBB2 and HER3/ERBB3 as well as several marker proteins for MVs. **B,** Detection of HER2 in whole-cell lysates and in MVs derived from noninvasive SK-Mel-173 cells with or without *PLK4* overexpression. Representative Western blots detecting HER2, PLK4, GAPDH (loading control for MVs) and α-tubulin (loading control for whole-cell lysates) are shown. **C,** Representative Western blots showing mild overexpression of HER2 in noninvasive SK-Mel-173 cells. β-actin was detected as a loading control. **D,** Measurements of MT growth rates in noninvasive melanoma cells after mild overexpression of *HER2* in the absence or presence of the irreversible HER2 kinase inhibitor canertinib. Scatter dot plots show average MT growth rates (20 MTs/cell, mean ± SD, *n* = 30, *t* test). **E,** Measurements of MT growth rates in the indicated invasive melanoma cells after treatment with DMSO (control) or the HER2 inhibitor trastuzumab. The scatter dot plots show average MT growth rates (20 MTs/cell, mean ± SD, *n* = 30, *t* test). **F,** 3D outgrowth of spheroids derived from invasive melanoma cells and treated with DMSO (control) or with the HER2 inhibitor canertinib. Left: Representative example images. Scale bar, 25 μm. Right: Quantification of the 3D outgrowth area of spheroids derived from the indicated invasive melanoma cells in the absence or presence of canertinib. The bar graphs show mean values ± SD (*n* = 19–27 spheroids, *t* test). **G,** Measurements of MT growth rates in noninvasive melanoma cells (SK-Mel-173) after treatment with MVs derived from noninvasive (SK-Mel-173) or invasive cells (SK-Mel-147 or SK-Mel-103) that were transiently treated with reversible (trastuzumab) or irreversible (canertinib) HER2 inhibitor. The scatter dot plots show average MT growth rates (20 MTs/cell, mean ± SD, *n* = 30, *t* test).

Next, we aimed to identify proteins that might contribute to MV-mediated paracrine signaling to increase MT dynamics in recipient cells. For this, we crudely isolated MVs from CM derived from *PLK4*-overexpressing or control transfected melanoma cells, that is, from cells with or without supernumerary centrosomes and increased MT growth (see: Experimental scheme in Supplementary Fig. S4A). Importantly, isolated MVs from *PLK4*-overexpressing cells showed full activity to increase MT growth rates when added to recipient cells while MVs from control transfected cells showed no such activity (Supplementary Fig. S4B). MVs isolated from three independent experiments were subjected to MS analysis and we identified a total of 4,421 proteins associated with the MVs from melanoma cells (Supplementary Table S1). Among them, many proteins known to be markers for MVs including GAPDH, CD9, CD63, CD81, TSG101, ADAM10, and Syntenin-1 (41) were identified in our analysis (Supplementary Table S1; please refer also to: microvesicles.org). These marker proteins were not enriched or lost in MVs from *PLK4*-overexpressing cells when compared with control cells (Fig. 5A; Supplementary Table S1), which is compatible with an equal enrichment of MVs in both settings.

However, upon data filtering and imputation we identified 172 proteins that were significantly enriched at least 1.5-fold in MVs derived from *PLK4*-overexpressing melanoma cells (Fig. 5A; Supplementary Table S1). Among these enriched proteins, we found the HER2/ERBB2 (1.96-fold enrichment) and HER3/ERBB3 (4.85-fold enrichment; Fig. 5A; Supplementary Table S1), both of which are known to form a functional heterodimeric growth factor receptor (42). HER2, in particular caught our attention, because it has been previously implicated as a key regulator of cancer cell invasion (19, 43–45) and, most importantly, it was found to be significantly activated upon the induction of supernumerary centrosomes to induce invasive acini in 3D breast epithelial cell cultures (20). We verified HER2 enrichment in MVs derived from noninvasive melanoma cells upon *PLK4* overexpression by Western blotting while the cellular levels of HER2 were not changed indicating that *PLK4* overexpression might cause enrichment of HER2 in MVs, but does not affect *HER2* expression (Fig. 5B). It is of note that we isolated MVs from constant numbers of growing cells (see: Materials and Methods) and did not directly quantify the number of MVs. Thus, it is formally possible that HER2 is either enriched in MVs or that more MVs with HER2 are shedded in response to *PLK4* overexpression. However, the equal amount of the GAPDH present in our MV preparations (Fig. 5B), which is among the most frequently detected constitutive proteins found in EVs (http://microvesicles.org/extracellular_vesicle_markers) and which we also detected as an unchanged constituent in our MS analysis (Supplementary Table S1) suggests that MVs might be enriched in HER2 content.

The detection of HER2 in MVs, together with the previous findings implicating a role of HER2 in cell invasion, prompted us to further explore the role of HER2 in MT growth regulation and melanoma cell invasion. First, we overexpressed *HER2* in noninvasive melanoma cells to mimic the condition in the SK-Mel-173 cells upon *PLK4* overexpression (Fig. 5C) and found a clear induction in MT growth rates as seen in invasive melanoma cells (Fig. 5D). Moreover, pharmacologic inhibition of HER2 kinase activity using the irreversible HER2 kinase inhibitor canertinib in *HER2*-overexpressing cells (Fig. 5D) or using trastuzumab (preventing HER2 dimerization and considered to be a reversible inhibitor for HER2 activation) in invasive melanoma cells lowered MT dynamics (Fig. 5E), together indicating that HER2 is involved in increasing MT growth rates in melanoma cells.

To investigate whether the HER2-mediated regulation of MT dynamics impacts on melanoma cell invasion as implicated from our results, we performed 3D spheroid invasion assays using the invasive SK-Mel-103 and SK-Mel-147 cells in the absence or presence of the HER2 inhibitor. In both cases, we observed a clear suppression of invasion activity upon HER2 inhibition (Fig. 5F) suggesting that HER2-mediated increased MT growth contributes to cell invasion in melanoma, which is in line with our results showing that inhibition of MT growth rates suppresses 3D invasion (Fig. 2E). HER2 inhibitor treatment did not grossly affect cell-cycle progression or induced cell death (Supplementary Fig. S5A).

Finally, we investigated whether HER2 might represent the relevant constituent in MVs that transfers the MT modulating activity onto recipient cells. We isolated MVs from noninvasive SK-Mel-173 or from invasive SK-Mel-103 and SK-Mel-147 cells and treated noninvasive SK-Mel-173 cells with the MVs. As shown in Fig. 5G, only MV isolated from invasive cells showed full activity to induce increased MT growth rates in the recipient cells while MVs from noninvasive cells had no effect. MVs from the invasive cells were then treated with the reversible HER2 inhibitor trastuzumab or with the irreversible HER2 kinase inhibitor canertinib followed by wash-off of the inhibitors to exclude that the inhibitors act directly on the recipient cells (Supplementary Fig. S4A). The washing step of MVs is also expected to (at least partially) remove trastuzumab from HER2 while canertinib would retain its inhibitory activity on HER2. In fact, live-cell measurements of MT growth rates in the recipient cells showed that treatment with canertinib grossly reduced MT growth rates while the trastuzumab wash-off had a weaker effect (Fig. 5G). Thus, HER2 represents a relevant constituent of MVs that transfer the MT dynamics inducing activity to adjacent cells to support cell invasion. Despite that *HER2* overexpression is sufficient to induce increased MT growth rates (Fig. 5D), it was not sufficient to induce 3D invasion in noninvasive melanoma cells (Supplementary Fig. S5B). These results are in line with the notion that increased MT dynamics is required, but not sufficient to induce melanoma cell invasion.

## Discussion

It is well established that supernumerary centrosomes are often detected in human cancer cells and can contribute to tumorigenesis by interfering with the proper execution of mitosis leading to whole chromosome missegregation and aneuploidy (23, 24). Independent of that, more recent work has revealed that supernumerary centrosomes can also support cell invasion, which is highly relevant during metastasis of cancer (19). Our work presented here, extends these findings by showing that supernumerary centrosomes cause increased MT growth rates in interphase, which are observed in invasive, but not in noninvasive melanoma cells. Moreover, we show that increased MT growth rates, rather than supernumerary centrosomes *per se* are required, but not sufficient to trigger melanoma cell invasion. Thus, supernumerary centrosomes and increased MT growth as a consequence thereof might represent relevant markers and mediators for cell invasiveness in melanoma cells. These results might also indicate that that cancer-associated aberrations other than amplified centrosomes can contribute to increased MT growth rates and thus, to cell invasion.

Supernumerary centrosomes clearly affect mitosis by mediating transient multipolar spindle intermediates, which contribute to whole chromosome missegregation and induction of aneuploidy (24). This is highly reminiscent to our previous results showing that increased MT growth rates during mitosis are typically seen in chromosomally unstable cancer and are responsible for whole chromosome missegregation in human cancer cells (29). Thus, there are remarkable parallels between supernumerary centrosomes and increased MT growth involved in chromosome missegregation (in mitosis) and in cell invasion (in interphase). It is currently not known how supernumerary centrosomes lead to abnormally increased MT growth rates in interphase, but enhanced MT nucleation from additional centrosomes might affect the homeostasis of tubulin or the availability of MT growth regulating factors. It was also suggested that supernumerary centrosomes are associated with increased ROS, which might also influence MT growth rates (20). However, oxidative stress was shown to decrease MT dynamics, at least in cardiomyocytes, which suggests that ROS might not be responsible for increased MT growth rates in invasive melanoma cells (46).

It is most intriguing that the presence of supernumerary centrosomes can contribute to non–cell-autonomous cell invasion. In fact, the Godinho lab showed that the induction of supernumerary centrosomes in breast epithelial cells and in pancreatic cancer cells promotes the secretion of soluble proteins and of proteins associated with EVs that contribute directly or indirectly to cell invasion in a paracrine manner (20, 21). This observation might provide a plausible explanation for the fact that at least a proportion of cancer cells with supernumerary centrosomes is maintained in a tumor cell population despite that fact that cells with supernumerary centrosomes suffer from reduced cellular fitness due to aneuploidy and high stress levels. For breast epithelial cells, it was shown that soluble proteins including IL8 rather than EVs are contributing to cell invasion (20). In contrast, pancreatic cancer cells with supernumerary centrosomes secrete EVs that activate cancer-associated stellate cells that, in turn, promote cell invasion of the cancer cells (21). However, the identity of the EV-associated proteins that activate stellate cells to promote invasion remain unknown. Nevertheless, supernumerary centrosomes seem to affect cell invasion by paracrine signaling through various means involving soluble as well as small and large EV-associated proteins. For invasive melanoma cells, we found that MVs rather than small exosomes or secreted soluble proteins are required to transfer the MT growth activity onto recipient cells to support invasion. However, this does not exclude the possibility that also soluble signaling proteins like IL8 might also contribute to cell invasion in melanoma. However, the main paracrine activity responsible for increasing MT growth in melanoma cells seems to be associated with MVs. Although we have not further clarified the cellular origin of the MVs carrying the MT growth inducing activity, our MS analysis indicated that they harbor several markers for MVs including CD9, CD63, CD81, ADAM10, Syntenin-1, GAPDH, and others (41). Centrifugation at lower speed (14,000 × *g*) was sufficient to remove the activity to increase MT growth from conditioned media and, *vice versa*, full activity was retrieved in low-speed centrifugation pellet fractions. This might indicate that larger MVs rather than smaller exosomes, which are typically pelleted only by centrifugation at >100,000 × *g* (41, 47), harbor the MT growth inducing activity.

Our MS analysis identified the growth factor receptor HER2/ERBB2 as a constituent enriched in MVs derived from melanoma cells with supernumerary centrosomes and increased MT growth rates. Because we have not quantified the number of secreted MVs we cannot exclude the possibility that supernumerary centrosomes might also trigger an increase on the number of HER2-positive MVs. HER2 is a member of the ERBB family of oncogenic growth factor receptors, which are frequently amplified, overexpressed, or mutated in human cancer (44). HER2 can form functional dimers with HER3/ERBB3 or with EGFR (42, 48), both of which were also found to be enriched in MVs from melanoma cells with supernumerary centrosomes. In agreement with this, HER2 has been detected on MVs from metastatic breast cancer and from non–small cell lung cancer (49–51). Thus, this might suggest that functional HER2/HER3 and/or HER2/EGFR dimers are incorporated into MVs and transferred onto recipient cells to trigger an increase in MT growth rates, which support cell invasion. This notion would be in line with substantial previous evidence that HER2 upregulation can trigger cell migration and invasion (19, 43–45) and it is also in agreement with the observation that HER2 is activated upon centrosome amplification and required for cell invasion in this context (20). Thus, HER2-associated growth factor receptors might act as regulators of MT growth rates to contribute to cell invasion. On the other hand, our results indicate that HER2 inhibition is more potent to suppress 3D cell invasion (Fig. 5F) when compared with conditions of suppressed MT growth (e.g., upon partial *CKAP5* depletion; Fig. 2E), which might suggest that HER2 can act through additional mechanisms to contribute to cell invasion. It is currently unknown how HER2 regulates MT dynamics. To our knowledge, there is no evidence for HER2 in direct MT binding or colocalization with MTs. However, because HER2 receptors can activate MAP kinase signaling, one could speculate that MAP kinases might contribute to the regulation of MT plus end growth. Interestingly, the oncoprotein and MT-associated protein Stathmin/Op18, which is well known to regulate MT dynamics is regulated by growth factors and ERK signaling (52), Hence, HER2-MAP kinase signaling might affect MT growth phenotypes through phosphorylation of MT-regulating proteins and Stathmin/Op18 might be one putative candidate.

An important question that remains yet unanswered is how supernumerary centrosomes and increased MT growth rates affect cell invasion. It seems plausible that altered MT organization and dynamics might affect polarized trafficking and signaling at the leading edge and into invasive structures as suggested earlier (9). Interestingly, it has also been shown that supernumerary centrosomes are associated with increased RAC1 activity, which can directly influence actin organization contributing to the formation of invasive structures and 3D cell movement. However, despite this interesting link it remains elusive how MTs can control activity of GTPases. It is possible that RAC1 specific GAPs or GEFs might be directly or indirectly regulated by dynamic MTs. It will be important for future research to address the link between MT dynamics and actin organization in more detail to understand how dynamic MTs contribute to cancer cell invasion.

## Supplementary Material

Figure S1 Figure S1 shows that low-dose Taxol or CKAP5 siRNA treatment does not induce cell cycle alterations or cell death while CKAP overexpression increases microtubule growth rates.

Figure S2 Figure S2 shows that CKAP5, STIL or PLK4 overexpression is not sufficient to significantly increase spheroid outgrowth in 3D matrices.

Figure S3 Figure S3 shows that conditioned media from invasive melanoma cells or from non.-invasive cells overexpressing PLK4 or STIL do not induce supernumerary centrosomes.

Figure S4 Figure S4 shows that microvesicles from non-invasive melanoma cells with PLK4 overexpression increase microtubule growth rates in recipient cells.

Figure S5 Figure S5 shows that HER2 inhibition does not affect cell cycle progression or cell death and HER2 overexpression is not sufficient to significantly increase spheroid outgrowth in 3D matrices.

Supplementary Video S1 The supplementary Movie S1 shows an example movie displaying the detection of EB3-GFP in a living interphase melanoma cell,

Supplementary Table S1 The supplementary Table S1 provides the results of the mass spectrometry results comparing protein content of microvesicles from melanoma cells with or without PLK4 overexpression.
