# Supplementary material for: Increased Microtubule Growth Triggered by Microvesicle-mediated Paracrine Signaling is Required for Melanoma Cancer Cell Invasion
Source: Cancer Res Commun. 2022 May 18;2(5):366–79. doi: 10.1158/2767-9764.CRC-22-0010 (PMC9981201; doi:10.1158/2767-9764.CRC-22-0010)
Supplement: Figure S1 — shows that low-dose Taxol or CKAP5 siRNA treatment does not induce cell cycle alterations or cell death while CKAP overexpression increases microtubule growth rates. [file crc-22-0010-s01.pdf]

Figure S1

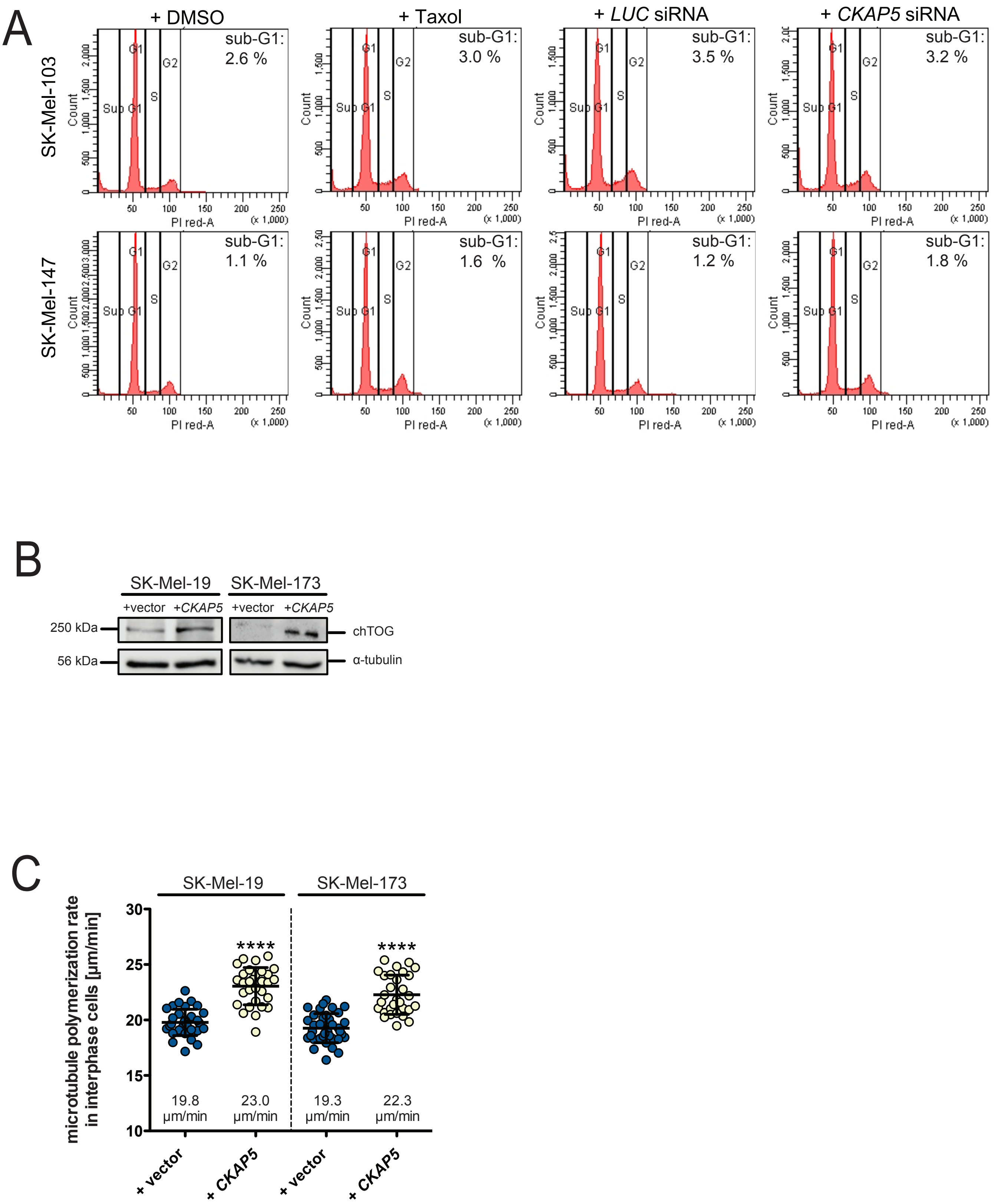

A
+ DMSO
+ Taxol
+ LUC siRNA
+ CKAP5 siRNA
SK-Mel-103
SK-Mel-147
sub-G1: 2.6 %
sub-G1: 3.0 %
sub-G1: 3.5 %
sub-G1: 3.2 %
sub-G1: 1.1 %
sub-G1: 1.6 %
sub-G1: 1.2 %
sub-G1: 1.8 %
Sub G1
G1
S
G2
Count
PI red-A
(x 1,000)
B
SK-Mel-19
SK-Mel-173
+vector
+CKAP5
250 kDa
56 kDa
chTOG
α-tubulin
C
SK-Mel-19
SK-Mel-173
microtubule polymerization rate in interphase cells [µm/min]
****
19.8 µm/min
23.0 µm/min
19.3 µm/min
22.3 µm/min
+ vector
+ CKAP5

Figure S1. Modulating microtubule growth rates in melanoma cells.

A, Representative FACS analysis of invasive melanoma cells with or without treatment with low dose Taxol or partial siRNA-mediated depletion of ch-TOG. Cells were treated as indicated for 72 h followed by FACS analysis detecting DNA content in three independent experiments. The proportion of cells with sub-G1 DNA content was quantified and is indicated for the given experiment. **B,** Representative western blots showing overexpression of *CKAP5* (ch-TOG) in non-invasive melanoma cells. α-tubulin was detected as a loading control. **C,** Measurements of microtubule growth rates in non-invasive melanoma cells after overexpression of *CKAP5*. Scatter dot plots show average microtubule growth rates (20 microtubules/cell, mean $\pm$ SD, n=30, *t*-test).
