# Supplementary material for: Increased Microtubule Growth Triggered by Microvesicle-mediated Paracrine Signaling is Required for Melanoma Cancer Cell Invasion
Source: Cancer Res Commun. 2022 May 18;2(5):366–79. doi: 10.1158/2767-9764.CRC-22-0010 (PMC9981201; doi:10.1158/2767-9764.CRC-22-0010)
Supplement: Figure S2 — shows that CKAP5, STIL or PLK4 overexpression is not sufficient to significantly increase spheroid outgrowth in 3D matrices. [file crc-22-0010-s02.pdf]

Figure S2

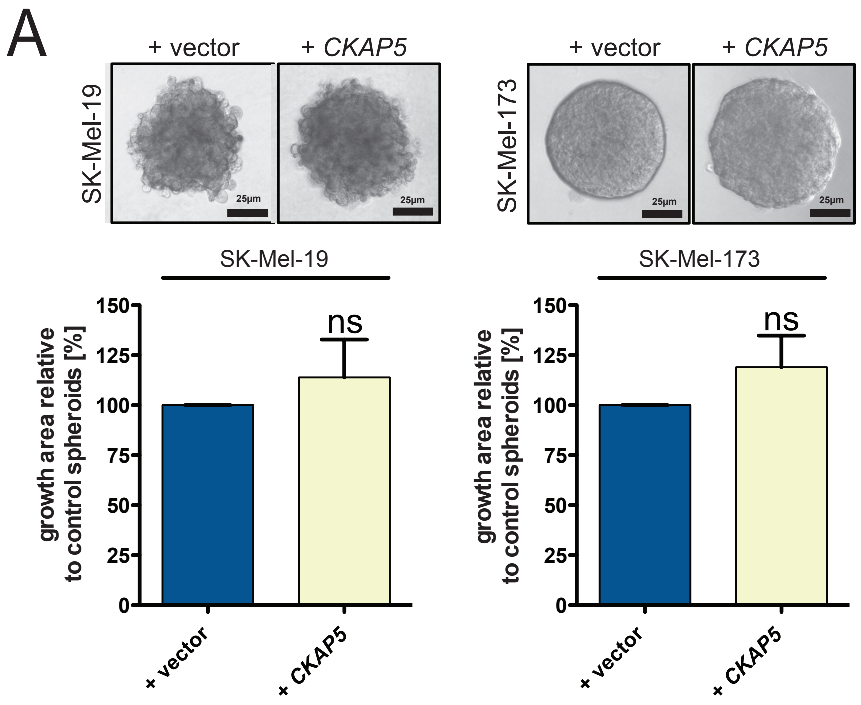

A
+ vector
+ CKAP5
SK-Mel-19
25µm
SK-Mel-173
SK-Mel-19
SK-Mel-173
growth area relative to control spheroids [%]
150
125
100
75
50
25
0
ns
+ vector
+ CKAP5

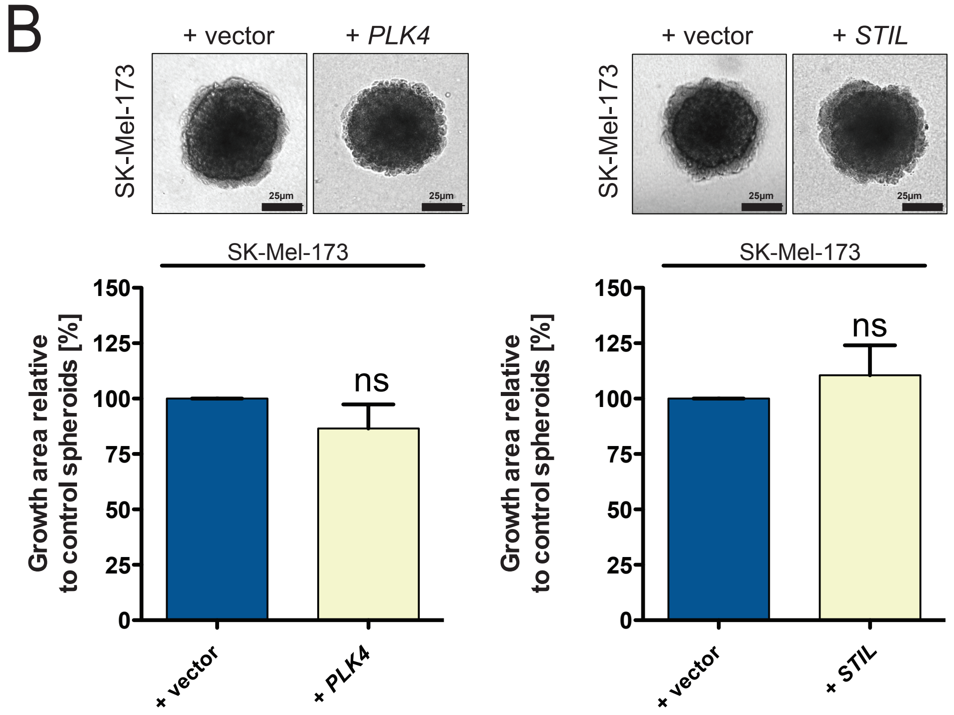

B
+ vector
+ PLK4
+ STIL
SK-Mel-173
25µm
Growth area relative to control spheroids [%]
150
125
100
75
50
25
0
ns
+ vector
+ PLK4
+ STIL

Figure S2. Increased microtubule growth rates are not sufficient to induce melanoma cell invasion.

A, Quantification of the 3D outgrowth area of spheroids derived from non-invasive SK-Mel-19 or SK-Mel-173 melanoma cells with or without increased microtubule growth rates induced by *CKAP5* overexpression. Representative images of spheroids grown for 48 hours are shown. Scale bar, 25 µm. The bar graphs show mean values ± SD (n=24-32 spheroids, *t*-test). **B,** Quantification of the 3D outgrowth area of spheroids derived from non-invasive SK-Mel-173 melanoma cells with or without increased centrosome numbers and increased microtubule growth rates induced by *PLK4 or STIL* overexpression. Representative images of spheroids grown for 48 hours are shown. Scale bar, 25 µm. The bar graphs show mean values ± SD (n=32 spheroids for PLK4; n=27 spheroids for STIL, *t*-test).
