# Supplementary material for: Increased Microtubule Growth Triggered by Microvesicle-mediated Paracrine Signaling is Required for Melanoma Cancer Cell Invasion
Source: Cancer Res Commun. 2022 May 18;2(5):366–79. doi: 10.1158/2767-9764.CRC-22-0010 (PMC9981201; doi:10.1158/2767-9764.CRC-22-0010)
Supplement: Figure S3 — shows that conditioned media from invasive melanoma cells or from non.-invasive cells overexpressing PLK4 or STIL do not induce supernumerary centrosomes. [file crc-22-0010-s03.pdf]

Figure S3

A

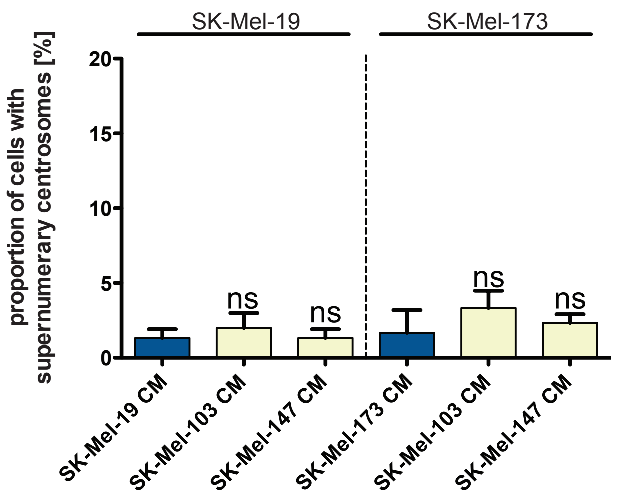

SK-Mel-19
SK-Mel-173
proportion of cells with supernumerary centrosomes [%]
20
15
10
5
0
ns
ns
ns
ns
SK-Mel-19 CM
SK-Mel-103 CM
SK-Mel-147 CM
SK-Mel-173 CM
SK-Mel-103 CM
SK-Mel-147 CM

B

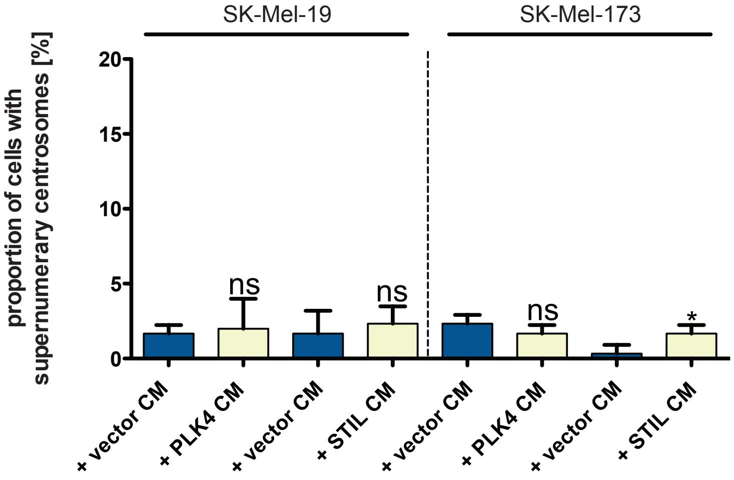

SK-Mel-19
SK-Mel-173
proportion of cells with supernumerary centrosomes [%]
20
15
10
5
0
ns
ns
ns
*
+ vector CM
+ PLK4 CM
+ vector CM
+ STIL CM
+ vector CM
+ PLK4 CM
+ vector CM
+ STIL CM

Figure S3. Conditioned media from invasive melanoma cells or from non-invasive cells overexpressing *PLK4* or *STIL* do not induce supernumerary centrosomes.

A, Quantification of the proportion of non-invasive SK-Mel-19 and SK-Mel-173 melanoma cells with supernumerary centrosomes upon treatment with conditioned media derived from non-invasive (SK-Mel-19, SK-Mel-173) or invasive (SK-Mel-103, SK-Mel-147) melanoma cells (mean ± SD, n=300 cells, *t*-test). **B,** Quantification of the proportion of non-invasive SK-Mel-19 and SK-Mel-173 melanoma cells with supernumerary centrosomes upon treatment with conditioned media derived from the same cells with or without *PLK4* or *STIL* overexpression (mean ± SD, n=300 cells, *t*-test).
