# Supplementary material for: Increased Microtubule Growth Triggered by Microvesicle-mediated Paracrine Signaling is Required for Melanoma Cancer Cell Invasion
Source: Cancer Res Commun. 2022 May 18;2(5):366–79. doi: 10.1158/2767-9764.CRC-22-0010 (PMC9981201; doi:10.1158/2767-9764.CRC-22-0010)
Supplement: Figure S4 — shows that microvesicles from non-invasive melanoma cells with PLK4 overexpression increase microtubule growth rates in recipient cells. [file crc-22-0010-s04.pdf]

Figure S4

A

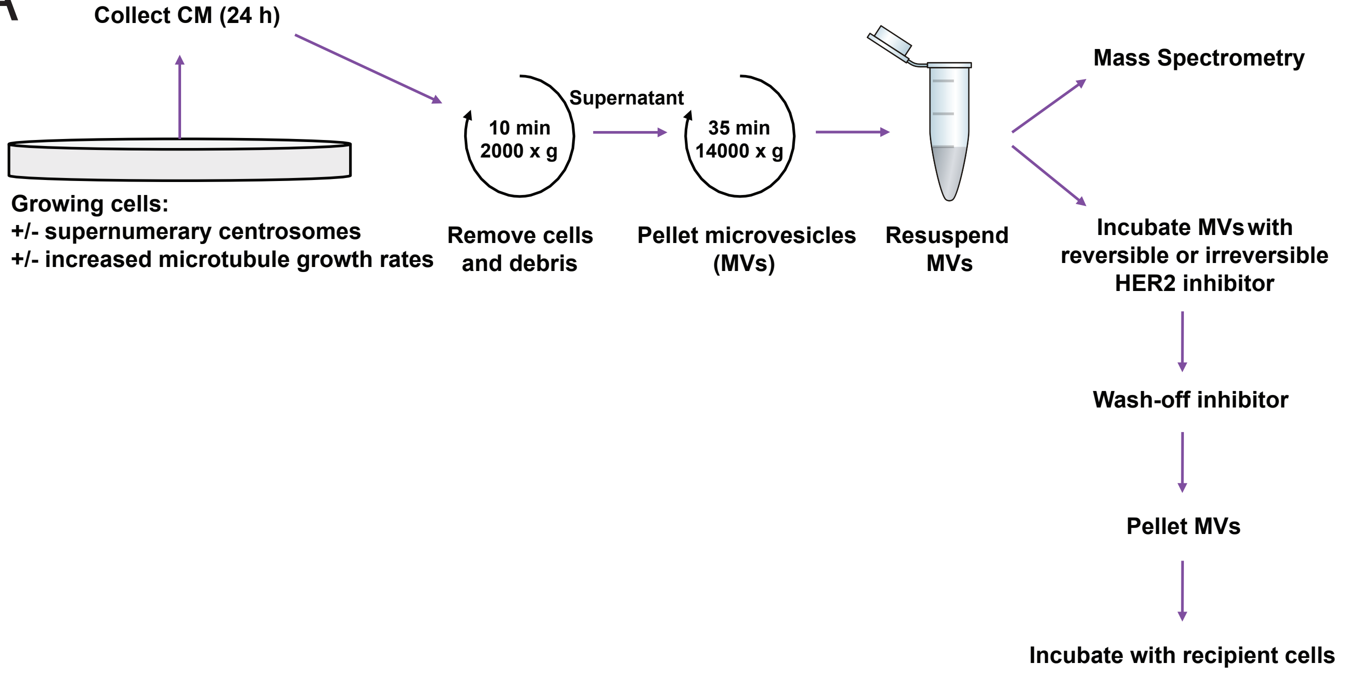
Collect CM (24 h)
Growing cells:
+/- supernumerary centrosomes
+/- increased microtubule growth rates
10 min
2000 x g
Supernatant
35 min
14000 x g
Remove cells
and debris
Pellet microvesicles
(MVs)
Resuspend
MVs
Mass Spectrometry
Incubate MVs with
reversible or irreversible
HER2 inhibitor
Wash-off inhibitor
Pellet MVs
Incubate with recipient cells

B

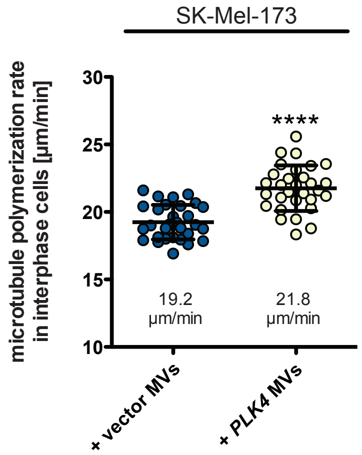
SK-Mel-173
microtubule polymerization rate
in interphase cells [µm/min]
30
25
20
15
10
****
19.2
µm/min
21.8
µm/min
+ vector MVs
+ PLK4 MVs

Figure S4. Microvesicles mediate the induction of increased microtubule growth rates. **A,** Isolation of microvesicles and HER2 inhibitor treatment scheme. **B,** Measurements of microtubule growth rates in non-invasive SK-Mel-173 melanoma cells after 2 h treatment with isolated microvesicles derived from the same cells with or without *PLK4* overexpression. Scatter dot plots show average microtubule growth rates (20 microtubules/cell, mean ± SD, n=30, *t*-test).
