# Supplementary material for: Increased Microtubule Growth Triggered by Microvesicle-mediated Paracrine Signaling is Required for Melanoma Cancer Cell Invasion
Source: Cancer Res Commun. 2022 May 18;2(5):366–79. doi: 10.1158/2767-9764.CRC-22-0010 (PMC9981201; doi:10.1158/2767-9764.CRC-22-0010)
Supplement: Figure S5 — shows that HER2 inhibition does not affect cell cycle progression or cell death and HER2 overexpression is not sufficient to significantly increase spheroid outgrowth in 3D matrices. [file crc-22-0010-s05.pdf]

Figure S5

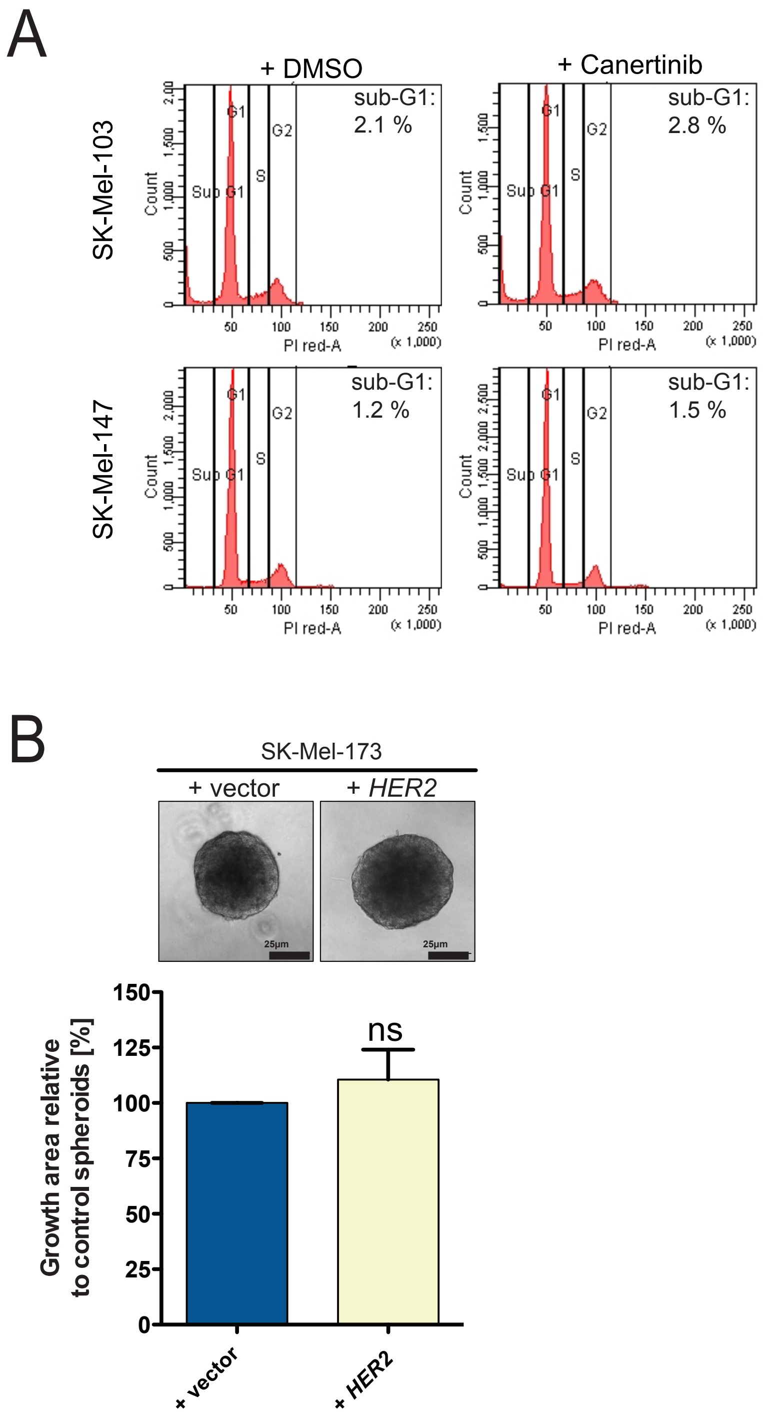
A
+ DMSO
+ Canertinib
SK-Mel-103
SK-Mel-147
sub-G1:
2.1 %
sub-G1:
2.8 %
sub-G1:
1.2 %
sub-G1:
1.5 %
Sub G1
G1
S
G2
Count
PI red-A
(x 1,000)
50
100
150
200
250
B
SK-Mel-173
+ vector
+ HER2
25µm
Growth area relative to control spheroids [%]
150
125
100
75
50
25
0
ns
+ vector
+ HER2

Figure S5. Microvesicles are not sufficient to induce increased cell invasion in non-invasive melanoma cells.

A, Representative FACS analysis of the indicated invasive melanoma cells with or without treatment with 1 µM Canertinib (HER2 inhibitor). Cells were treated for 72 h followed by FACS analysis detecting DNA content in three independent experiments. Cells with sub-G1 DNA content were quantified and are indicated for the given experiment. **B,** Quantification of the 3D outgrowth area of spheroids derived from non-invasive SK-Mel-173 melanoma cells with or without *HER2* overexpression. Representative images of spheroids grown for 48 hours are shown. Scale bar, 25 µm. The bar graphs show mean values ± SD (n=42 spheroids, *t*-test).
